# Development of a Business Model Resilience Framework for Managers and Strategic Decision-makers

**DOI:** 10.1007/s41471-022-00135-x

**Published:** 2022-07-21

**Authors:** M. Radic, P. Herrmann, P. Haberland, Carla R. Riese

**Affiliations:** Fraunhofer IMW, Leipzig, Germany

**Keywords:** Resilience, Framework, Business model, Business model resilience, Covid-19, SME, D22, D24, E32, H12, L14, L23, L25, M11, M12, M51

## Abstract

Following the massive impact of the Covid-19 pandemic on the global economy and on small and medium-sized enterprises (SMEs) in particular, the concept of resilience has experienced a renaissance. As an organizational concept, business model resilience describes the extent to which an organization can maintain or quickly recover its value proposition despite unexpected current or future disruptions (Palzkill-Vorbeck 2018). Although research has been conducted in this area for decades, there is still a lack of a unified framework that brings together the findings from research and links them to organizational practice. The paper addresses this gap by developing a framework for business model resilience and demonstrating its practical relevance for organizational performance during the Covid-19 pandemic in 2020. The framework includes 11 factors that characterize the resilience of an organization’s business model. For managers and decision-makers, the framework is an opportunity to assess and improve the resilience of their organizations. For researchers, the framework is an important foundation for transferring the concept of business model resilience into organizational practice.

## Introduction

After its first discovery in December 2019, the Covid-19 virus has since spread worldwide, causing a global pandemic with massive economic impact (Federal Ministry of Health 2022). On a macroeconomic level, global GDP fell by 3.3% to −3.4% in 2020 and still has not fully returned to its pre-crises level (OECD 2021). The German economy declined by 4.6% in 2020 and grew by 2.7% in 2021 (Federal Statistical Office 2022). In addition, the Association of German Chambers of Industry and Commerce (2022) estimates the impact of the Covid-19 pandemic on the economic output in Germany in 2020 and 2021 at 400 billion euros. On a microeconomic level, organizations had to react to numerous challenges in the short term. The most notable challenges include reduced incoming orders, delivery problems or production stops (Federal Statistical Office 2021). With the introduction of vaccinations, organizations have become more optimistic that the economic pressure from the Covid-19 pandemic will ease in 2021 (Allgäuer Zeitung 2020; Handelszeitung 2020; Unternehmerverband 2020). However, at the time of writing this paper in February 2022, ongoing mutations continue to keep pressure on the economy. Most of the mentioned challenges still remain, especially supply chain restrictions. Managers do not expect their organizations to recover before the end of 2022 (DIHK 2022; Hinze 2021).

Against this background, the concept of resilience is becoming more important. Applied to organizations by Sutcliffe and Vogus (2003), it helps to understand why similar organizations perform differently when dealing with the same crisis (Thun-Hohenstein et al. 2020). We use the business model resilience perspective, which describes a company’s ability to handle unforeseeable events and crises, such as the Covid-19 pandemic, and maintain its value proposition during the crisis (Palzkill and Augenstein 2017). However, even though the concept of resilience has been used in an organizational context for years, it remains unclear what exactly the factors are that distinguish organizations with high resilience from those with low resilience.

This study is the first to provide empirical evidence of the practical relevance of the concept of business model resilience. Although several authors have addressed the topic from a theoretical (e.g. Davoudi et al. 2013; Gibson and Tarrant 2010; Palzkill and Schneidewind 2014; Wieland and Durach 2021) as well as from a practical (e.g. Cronenberg 2020; Drath 2018; Stephenson 2010) perspective, no study has yet combined both theoretical and practical considerations in one holistic framework supported by empirical data. Therefore, the objectives of this study are:

1. Develop a framework for business model resilience that is based on existing research
2. Demonstrate the practical relevance of the framework by linking it to business performance indicators

By achieving both objectives, the study is an important step towards operationalizing the concept of business model resilience and making it practical for managers and decision-makers. The framework and its associated items can be used to assess the resilience of a company’s business model and serve as a basis for targeted measures to improve it. Thus, it is an important tool for preparing for unexpected crises and will ultimately help organizations to resist economic setbacks. The paper also closes an important gap in research by aggregating distributed findings on business model resilience into a unified framework and validating it in practice. The framework presented provides a holistic overview of business model resilience, that is theoretically and empirically backed. It provides an opportunity for researches to further investigate the resilience factors presented and map them to the framework research can be mapped to the framework to better understand the impact and interplay of the resilience factors presented.

The paper describes the development of a longlist of items to measure all 13 factors of business model resilience derived from a systematic literature research. In a survey with managers, the list was reduced to 27 items using a combination of quantitative and qualitative methods. Using this set of items, which cover 11 factors of business model resilience, we demonstrate that organizations scoring high on business model resilience performed significantly better than organizations scoring low on business model resilience during the Covid-19 pandemic in 2020.

The paper is structured in the following way: Chapter 2 explains the theoretical background of business model resilience and presents the findings from a systematic literature research that serves as the theoretical basis for this study. Chapter 3 outlines the research design of the empirical study in the manufacturing industry in Saxony (Germany). Chapter 4 provides an overview of the results of the study, structured by the two research objectives mentioned above. Chapter 5 condensates the findings in the Fraunhofer IMW business model resilience framework. The study concludes with an appreciation of the limitations of the study and an outlook for future research.

## Theoretical Background

The conceptualization of resilience varies widely across research disciplines and the perspectives taken. However, “in order for resilience to be a useful and valid concept, it is necessary to have a solid understanding of the origin of the concept and how it is defined, by which variables it is determined, and how it can be assessed, maintained and improved over time” (Linnenluecke 2017). The following chapter will provide the reader with a theoretical and definitional overview of the concept of business model resilience used in this paper. Chapter 2.1 serves three purposes. First, a brief introduction to the historical foundation of the concept will be given. Second, four different levels of resilience that are widely used in current research and practice will be presented (Brink et al. 2021; Cronenberg 2020). Third the concept of business model resilience will be defined and differentiated from risk management,business continuity management and organizational agility. Based on this overview, chapter 2.2. describes a systematic literature review conducted to identify all relevant factors to describe business model resilience.

### Definition of Relevant Terms

Historically, the term resilience comes from the Latin word *resilire* (to rebound, to bounce back) and describes the elastic deformation of a body that returns to its original state after being subjected to force. In the 1970s, the term was applied to humans by psychologist John Block (Thun-Hohenstein et al. 2020). The following years, research was mainly driven by psychology, focusing on resilience on an individual level. It describes the ability to cope with unpredictable crises through reflection, to recognize one’s own relationship with the environment, to identify precautionary measures, to install appropriate protective mechanisms and to relocate and regenerate after a crisis (Baltes and Freyth 2017; Linkov and Trump 2019). Accordingly, five phases of resilience have been formulated: Prepare, protect, prevent, respond and recover (Thoma et al. 2016). The concept has been applied in practice for example to improve work design (Hartwig et al. 2016). Resilience has also been studied from a social-ecological perspective and is closely intertwined with sustainability research. In this context, it describes the capacity of a system to handle both extreme disturbances as well as persistent stress (Marchese et al. 2018; Roostaie et al. 2019). By considering environmental, social, and economic systems, a broad scope is opened with the potential to better prepare for complex and complicated systematic issues.

Applying the concept of resilience to organizations, Cronenberg (2020) proposes three different levels of analysis. The individual level, the team level and the organizational level. Brink et al. (2021) adds the environmental level. The individual level focuses on resilience on a personal level (i.e. the historical perspective on resilience), such as whether individuals have the resources they need to respond to a crisis. The team level considers how individuals interact with each other, e.g. whether there is a culture of communication that enables flexible adaption to changing circumstances. At the organizational level, it is about the resilience of different organizational areas, e.g. whether the production capacity can be managed to cope with changing demands. Finally, the environmental level considers interactions with other organizations and the broader cultural or legal conditions under which an organization operates. It becomes obvious that the perspectives on resilience described above (psychological, social-ecological, organizational) are not mutually exclusive. For example, from an organizational perspective, the individual level of resilience provides opportunities to connect with the psychological perspective, the environmental level with the social-ecological perspective. This has implications for both researchers as well as practitioners. For researchers, there is great potential for synergy by bringing together findings from different perspectives. For practitioners, it implies that if the objective is to minimize adverse effects of a potential crisis, different levels of resilience must be considered.

Having outlined the different levels that ought to be considered when looking at organizational resilience, the concept itself has yet to be defined. In the literature, various definitions of resilience in a business context exist. There is an ongoing conceptual discussion on how to define the concept and how it differs from related managerial concepts (e.g. organizational flexibility, organizational change capacity, organizational adaptive capacity or organizational buffering capacity; Hillmann and Guenther 2021). While the paper does not aim to disentangle similarities and dissimilarities with other managerial concepts, we follow the authors’ request in gathering more evidence on what constitutes organizational resilience. Thus, we propose to adapt the perspective of business model resilience as a working definition for our study. We use this terminology to mark the perspective through which resilience should be reflected in organizations: It is a strategic topic that should be addressed by managers and decision-makers because, similar to a “regular” business model, it ultimately determines the future of an organization by linking corporate strategy to operations (Palzkill-Vorbeck 2018). We define business model resilience as the ability of an organization to sustain its value proposition despite unexpected current and future disruptions (Palzkill and Augenstein 2017). This ability can manifest at the individual level, team level, organizational level, or environmental level (Cronenberg 2020; Drath 2018). Through short-term adaptability or agility, a resilient business model enables an organization to return to a predefined starting position as quickly as possible after a disruption (Günther et al. 2007). Lessons learned from disruptions that have occurred are used to proactively anticipate potential risks (risk management) as well as to integrate long-term benefits from changing environmental conditions into the system, e.g., through learning or through innovation (Drath 2018; Duchek 2020; Gibson and Tarrant 2010; Marks 2015).

A question that often arises is to what extent business model resilience is a truly new concept and how it differs from concepts already applied in managerial practice.

Risk management consists of risk assessment, risk communication, and risk processing (Engemann and Henderson 2012). It presents itself as a process that identifies risks in advance and makes them tangible or reduces them in order to make systems less vulnerable to crises that occur. The aim is to make the system safe, to reduce accidents and errors, and to reduce the impact of disruptions in organizations through organizational measures (Hartwig et al. 2016). Risk management thus has a preventive character and provides valuable information for an overall business continuity management (Engemann and Henderson 2012). However, it only covers a fraction of the much broader concept of resilience, as it is mainly concerned with handling anticipatable risks. Schäffer (2020) sees strategic risk management as an essential component of resilience, but also does not see it as the only lever for strengthening corporate resilience.

Business continuity management, on the other hand, describes the active planning, control and safeguarding of the long-term continuity and success of an organization. This is achieved by realizing organizational resilience to events that damage the organization (Eisele 2020). It is to be understood as a holistic management program with perspectives on the relevant stakeholders, the environment, the organization’s reputation, the brand, and the value creation activities. It does not focus on preventive aspects, but on the consequences of crises. This means that risk management and business continuity management should be considered as complementary concepts (Engemann and Henderson 2012). However, both risk management as well as business continuity management lack a holistic view of corporate resilience that maps all phases of resilience development, including the phase of learning from crises (vgl. Hartwig et al. 2016).

Finally, the term organizational agility is used to describe the ability of an organization to manage change proactively (Miceli et al. 2021). Based on a systematic literature review Gligor et al. (2019) point out conceptual overlap as well as differences between agility and resilience. The main difference concerns that agility is more focused on the ability to quickly change directions while resilience is more concerned with the ability to resist/survive disruptions. However, Miceli et al. (2021) argue that agility “builds a strategic dimension of resilience […]. It includes the notion of the speed of the organization’s response to change”.

Therefore, we conclude that risk management, business continuity management and organizational agility have some conceptual overlap with business model resilience. However, the latter can be considered as a more holistic approach for managers and decision-makers to look at how their organization is capable of maintaining its value proposition despite unexpected crisis by considering the individual, team, organizational and environmental level. The question what exactly are the key factors that characterize a resilient business model will be answered through a systemic literature review in the following chapter.

### Systematic Literature Research on Business Model Resilience

Although assessing, managing, and improving organizational resilience from a managerial perspective (i.e. business model resilience) has received increasing interest in the research landscape, there is a lack of a systematic overview of all factors that should be considered. Existing papers either focus on partial aspects (e.g. Kashav et al. 2019) or develop frameworks that seem plausible in practice but often lack a sound theoretical background (e.g. Schäffer 2020). We therefore conducted a systematic literature review to capture all factors discussed in the literature on business model resilience. First, we identified relevant papers by applying the established PRISMA method (Moher et al. 2009). Since none of the identified papers were able to provide an exhaustive overview of factors that determine an organization’s resilience from a managerial perspective, we used an inductive approach following Kuckartz (2016) and Mayring (2000) to condensate the information extracted into 13 factors of business model resilience. The methodology is briefly explained below, followed by a detailed explanation of the factors that have been identified.

#### Methodology

**Fig. 1:**
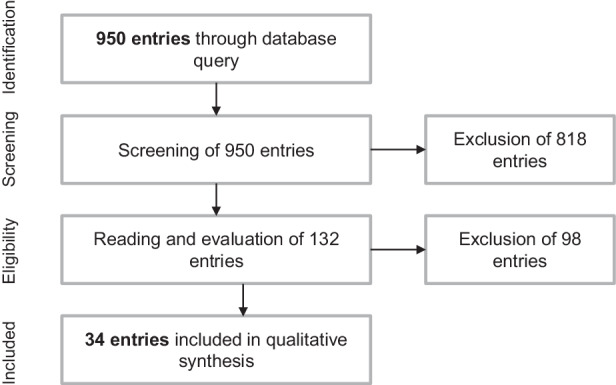
Flow chart of systematic literature search

As a first step, we applied the PRISMA methodology (Moher et al. 2009) to identify relevant papers. Following the steps described below, a total of 34 papers have been identified for further analysis (see Fig. 1).

- Identification
  In advance of the search, an individual search for optimal keywords was carried out. Subsequently, the databases EBSCO and the search portal of the Fraunhofer-Gesellschaft (E-Lib) were used for the search query.
  Eight queries were conducted at both search portals with the keywords resilience* + value creation/resilience* + business models/resilience* + organization/resilience* + cooperation/resilience* + value chain/resilience* + value networks. In total, the queries yielded 950 entries.
- Screening
  We screened the abstracts of the 950 entries to ensure that they are not redundant, that resilience is the core topic of the publications, that they examine resilience from an economic perspective (rather than from the perspective of other disciplines, such as individual-centered psychological research), and that they are related to business model resilience. Of the 950 entries, 818 were deemed to have little to no relevance and are not considered further.
- Eligibility
  The remaining 132 documents were analyzed intensively. Since the objective of the paper is to link business model resilience to organizational practice, we selected papers that empirically measure/operationalize business model resilience. We excluded another 98 documents that did not meet this criterion.
- Included
  The remaining 34 documents were included for in-depth qualitative analysis.

None of the 34 included papers included for analysis provided an exhaustive overview of factors that determine an organization’s resilience from a managerial perspective. For example, Drath (2018) proposes 14 factors, represented by 46 items, to assess an organization’s resilience. However, the set is missing relevant content as has been pointed out by other authors such as flexibility of the business model or the extent to which products are matched to customer’s needs (Palzkill-Vorbeck 2018; Flüter-Hoffmann et al. 2018). Other authors propose a compelling framework that is hard to put in to practice as the factors are not self-explanatory from a managerial perspective. For example, Palzkill-Vorbeck (2018) proposes to transfer established dimensions of resilience from a social-ecological perspective (precariousness, latitude, resistance, panarchy; see e.g. Walker et al. 2004) to business model resilience. However, these categories are not suitable for implementation into organizational practice as they do not point towards concrete organizational levers which improve business model resilience.

**Fig. 2:**
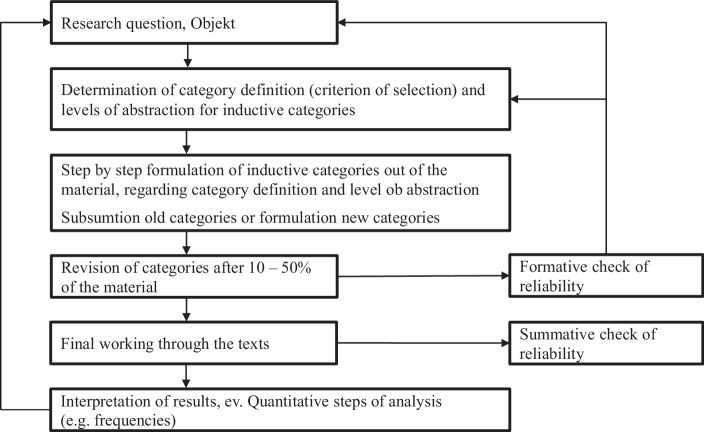
Step model of inductive category development (Mayring 2000)

We therefore, decided to extract business models resilience factors using an inductive approach (Kuckartz 2016; Mayring 2000). Fig. 2 provides an overview of the steps suggested by Mayring (2000). The objective of the qualitative content analysis was to identify an exhaustive overview of abstract categories that determine business model resilience and are self-explanatory from a managerial perspective in providing guidance for concrete levers that can be used to increase business model resilience (step 1 of the process outlined by Mayring 2000). As a second step, we specified the level of abstraction required for the categories (step 2) by extracting all categories provided in the literature identified that fulfill the criteria of being self-explanatory from a managerial perspective (e.g. supply chain; e.g. supply chain; Kashav et al. 2019). The level of abstraction depicted by this set of levers served as reference for the desired level of abstraction of the business model resilience categories extracted from the inductive analysis. We then extracted all items provided in the literature to assess business model resilience as primary source for the qualitative content analysis. We came up with a longlist of 130 concrete items to describe business model resilience (e.g. “We understand how we are connected to other organizations and actively manage those links.”; “Our organization understands the minimum level of resources it needs to operate successfully”; (Resilient Organisations Ltd 2014; Stephenson 2010). Following step 4 provided by Mayring (2000) and the procedure described by Elo and Kyngäs 2008), the four researchers involved in the study grouped together items that seem to belong to the same higher order category (e.g. “We have multiple suppliers/buyers to avoid the supplier/buyers disruptions”; Chowdhury and Quaddus 2017; “Our organisation has a good understanding of how quickly we would be affected if one of our larger customers or suppliers went out of business”; Stephenson 2010). Through this process, 16 categories were identified in an initial step. We named each category using content-characteristic words (e.g. “Supply chain”; see again Elo and Kyngäs 2008). Following the iterative process described by Mayring (2000) the research team went through the process of grouping items together and naming the category according to the items included several times until consensus was reached on a set of categories that provide a comprehensive description of business model resilience through subsuming items of similar content within each category while maximizing overlap between categories. We consider these categories to be factors of business model resilience. The 13 identified factors are described in detail below.

#### Results

By conducting the systematic literature research described above, 13 factors were identified that describe business model resilience. These factors include Crisis management, Corporate culture, Customer focus, Digitization, External collaboration, Finance, Human resources, Innovation, Leadership, Product focus, Strategy, Supply chain and Value proposition. The factors are explained in detail in the following.

##### Crisis Management (e.g. Duchek 2020; Gelbmann and Peskoller 2016)

Resilient organizations should always monitor internal and external developments, track and anticipate risks, and try to counter them with specific plans. In addition, the skills of people and structures must be developed and tested in order to be able to cope with challenges through flexibility and stability.

##### Corporate Culture (e.g. Asadzadeh et al. 2020; Azadeh et al. 2017)

The Corporate culture is noticeable internally to all employees and managers and externally to suppliers, customers, and other partners. It is the catalyst that enables the implementation of mission, vision and strategy, ensures organization-wide learning and, in times of crisis, maintains the cohesion, commitment and motivation of employees through sense-making and communication.

##### Customer Focus (e.g. Cabral et al. 2012; Carayannis et al. 2014)

Customer wishes change over time. They become more specific, fit more into lifestyles, have to be fulfilled promptly, and include the desire for high quality. At the same time, they need to be fulfilled in a cost-effective manner. Some of these needs persist during crises (e.g. ensuring toilet paper during the Covid-19 pandemic), while others evolve rapidly (e.g. providing masks during the Covid-19 pandemic). Resilient organizations can quickly identify and respond to these needs, but this also means that they have identified and established the right channels to engage with customers.

##### Digitization (e.g. Flüter-Hoffmann et al. 2018; Rapaccini et al. 2020)

Digitization is a driving force for every organization. It must be used even more in the context of resilience because it enables more efficient communication between people and between people and machines. It simplifies processes and enables completely new network-like structures inside and between organizations, for example in the context of agility, and is therefore indispensable in crises.

##### External Collaboration (e.g. Gimenez et al. 2017; Jones 2015)

Collaboration with external partners serve as resources that organizations can draw on in times of crises. They have to be established and nurtured in times of non-crisis and increase the security of organization. While the investment seems to be limited, the pay-off shows when organizations can rely on the collaborations built.

##### Finance (e.g. Lee et al. 2013; Schäffer 2020)

Revenues and expenses must be calculable at all times for organizations within the entire value chain. In the context of resilience, it is important to have financial leeway on the one hand and to establish different and flexible revenue models for products and services on the other hand. Both aspects make organizations well equipped to meet challenges in times of crisis.

##### Human Resources (e.g. De Carvalho et al. 2012; Lengnick-Hall et al. 2011)

Employees are the essential element of an organization. They are recruited, trained and developed in a targeted manner and increasingly take on responsibility. Especially in a crisis, organizations need to be able to rely on their commitment to the organization and their sovereignty to do their work in such a way that any failures or setbacks can be absorbed by them.

##### Innovation (e.g. Carayannis et al. 2014; Lee et al. 2013)

Innovation ensures technological progress throughout the entire value creation process, taking into account developments in the organization’s environment. In periods of crisis, innovations are necessary in order to react quickly to changing customer and societal needs. To be resilient, organizations must therefore develop and test innovation processes in non-crisis times. This is the only way innovations can create stability in times of crisis.

##### Leadership (e.g. Cantu et al. 2021; Hoffmann 2017)

Managers play a central role in the development of resilience. Among other things, they are responsible for the optimal use of human resources and thus for promoting knowledge, competencies, a sense of responsibility and independence. In this context, a balance must always be maintained between demanding and encouraging with regard to efficient achievement of the organizational goal and the costly development of resilience-promoting skills.

##### Product Focus (e.g. Kristianto et al. 2017; Thomas et al. 2016)

A key factor in periods of crisis is ensuring the delivery of products and services. For this purpose, all production-related information must be collected in the organization and be available at all times in order to adequately maintain production capacities and ensure delivery. In addition, a comprehensive product portfolio offers the security that individual products and services will be purchased or used even in times of crisis.

##### Strategy (e.g. Buchholz and Knorre 2012; Schäffer 2020)

The strategy of an organization is fundamentally designed as a permanent process. Resilient strategies enable flexible adaptation of the strategy in times of crisis in order to be able to adequately meet the specific challenges. This includes, for example, strategies in the area of securing the supply chain and/or in the area of customer loyalty.

##### Supply Chain (e.g. Chowdhury and Quaddus 2017; Porzig 2014)

An organization must decide how to structure its supply chain. In doing so, resilience is a cost factor, because in order to be able to avoid delivery failures, safeguards must be established. This means that the structure of suppliers must be changed with regard to specific aspects so that supplies reach the organization reliably through resilient supply chains.

##### Value Proposition (e.g. Palzkill and Schneidewind 2014; De Rosário Cabrita et al. 2016)

Even in times of crisis, organizations must maintain their value proposition to customers. To achieve this, flexibility and stability must be balanced in the relevant areas of the business model (cf. Osterwalder 2004).

**Fig. 3:**
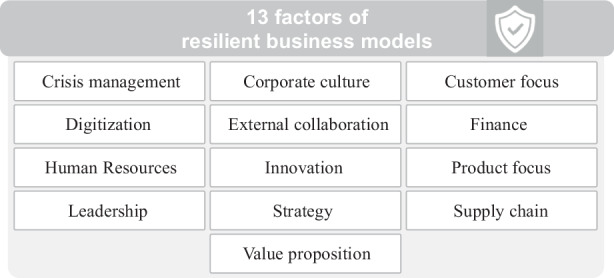
13 factors identified through systematic literature analysis

Fig. 3 illustrates the 13 business models resilience factors described above. It becomes evident that managing and improving business model resilience is a broad topic that spans across different departments in an organization (e.g. marketing, finance, human resources, strategy). Although the proposed factors have been systematically derived from the literature, the question of practical relevance remains to be answered. Hence, an online survey was conducted with the objective to validate the factors in practice and to link them to organizational performance during crisis periods.

## Research Design

Following the systematic literature described above, an empirical study has been carried out with two objectives. First, to develop a framework for business model resilience that is theoretically based on the results of the systematic literature review described above but condensed to a set of factors that are most relevant from a practitioner’s perspective. Second, to demonstrate the practical relevance of the developed framework. To be practical for managers and decision-makers, a resilient business model should have some organizational benefit, i.e. a better performance in crises. To answer both questions, we conducted an online survey with the structure shown in Table 1. The core sections B and C will be explained in more detail below.

**Table 1:** Structure of questionnaire

Section	Content
*A) Descriptives*	Industry
	Organization size: Revenue, number of employees (FTE)
	Organization location
	Department of respondent
*B) Performance during Covid-19 pandemic in 2020*	Overall impact of Covid-19 pandemic in 2020
	Revenue development 2020 vs. 2019
	Impact of Covid-19 pandemic on different organizational areas
	Internal organizational aspects that helped or hindered the management of the pandemic
*C) Business model resilience questionnaire*	Assessment of resilience scale

Section B “Performance during Covid-19 pandemic in 2020” mainly contains the dependent variables as well as some control variables. The three dependent variables used in the study are described in Table 2. Additionally, respondents were asked to name up to three internal aspects that have been beneficial or limiting when dealing with the Covid-19 pandemic. This non-mandatory question was included to qualitatively ensure that the literature-based questionnaire covers all relevant aspects of business model resilience.

**Table 2:** Description of dependent variables used in questionnaire

Dependent variable	Question	Scale
*Overall impact of Covid-19 pandemic on the organization in 2020*	All in all, how much of an impact did the Corona crisis have on your business in 2020?	7‑point Likert, 1: Strong negative impact 7: Strong positive impact
*Revenue development 2020 vs. 2019*	How did your revenue develop in 2020 compared to 2019?	9‑point Likert, 1: > 50% revenue decline 9: > 50% revenue growth
*Impact of Covid-19 pandemic on 13 different organizational areas*	How has the Corona crisis affected the following areas of your company? (Question was asked separately for 13 different areas.)	7‑point Likert, 1: Strong negative impact 7: Strong positive impact

To create the business model resilience questionnaire (section C), we used the set of 130 items extracted from the systematic literature research described in chapter 2.2. The items were scanned separately by two researchers and reduced to be mutually exclusive and collectively exhaustive. We generated a longlist of 54 items for the questionnaire, covering all 13 resilience factors derived from the systematic literature research. The questionnaire contained items worded as statements, such as “In our company, new ideas are tested quickly.”. Respondents were asked to rate the statement on two different dimensions. The first dimension is a self-assessment of the organization: “To what extent does this statement apply to your company?” (5-point Likert scale, 1—I strongly disagree; 5—I strongly agree). This dimension measures the organizations’ self-assessment in business model resilience in the respective item. The second dimension assesses whether respondents consider the item useful for capturing business model resilience: “To what extent do you consider the statement useful for dealing with crises such as the Corona pandemic?” (5-point Likert scale, 1—strongly disagree; 5—I strongly agree).

We programmed the survey with the free and open-source online survey application LimeSurvey. The target group were managers and decision-makers from SMEs in Saxony with an industry focus on the manufacturing sector (automotive supplier, machinery and plant engineering, medical technology and products, microelectronics). The survey took around 20 min to complete. As an incentive, respondents had the opportunity to win one of ten free mini projects following the survey. The mini projects aim to identify organization-specific measures to improve the resilience of their business model. Respondents could also choose to receive the survey results by mail. Three different phases aimed to ensure response rates. In the first phase, the survey link was sent out by a local multiplicator for economic development of SMEs in Saxony to a mailing list with over 2000 contacts. In a second phase, reminder emails were sent and social media channels (LinkedIn, Twitter) were also activated. In the third phase, phone calls were conducted to secure responses. In all three phases, compliance with General Data Protection Regulation (GDPR) was ensured. The online survey was live from July to October 2021.

## Results

The following chapter will outline the results of the online survey conducted with SMEs in Saxony. First, we will present descriptive results of the sample considered for the analysis. Secondly, we will show how we condensed the longlist of items and factors that we extracted from the systematic literature analysis to a set of items and factors that are most relevant from a practitioner’s perspective—what we call the business model resilience framework. Lastly, we demonstrate the practical relevance of this framework by linking business model resilience scores with organizational performance indicators during the Covid-19 pandemic in 2020.

### Descriptives

We were able to consider 67 responses for the analysis, corresponding to a response rate of around three percent. According to our experience in the field, this is an average response rate, especially when only managers and decision-makers are addressed. The main reason for organizations not to responding was a lack of time on their end. Organizations from the microelectronics sector mentioned during the follow-up phone calls we conducted that they had full order books and no capacity to complete the questionnaire. Some organizations had difficulties in understanding the concept of business model resilience and felt that it was not of practical relevance for them. Due to the evenly spread dropouts across the questionnaire, the survey construction itself is no reason for the low response rate. In addition, the lack of incentives was no reason for the low response rate, as the majority of respondents agreed to stay in contact regarding the survey results and the topic of business model resilience.

Table 3 shows size of the respondents’ organizations based on the EU classification (European Union 2003). About 80% of the respondents are small or medium-sized organizations. Almost 15% are micro enterprises with less than 10 employees, and 9% are large organizations.

**Table 3:** Organization size by EU classification

Industry	Number of organizations	Share (in %)
*Micro enterprises*	10	14.9
*Small enterprises*	26	38.8
*Medium-sized enterprises*	25	37.3
*Large enterprises*	6	9.0
*Total*	**67**	**100.0**

Table 4 describes the industry split of organizations included in the study. The low number of responses in microelectronics is likely due to the market dynamics being present at the time the survey was launched and bound the capacities of many organizations. On the other hand, the relatively high number of responses in machinery and plant engineering can be explained due to the local market structure—the segment is the largest from the industries included in the survey.

**Table 4:** Distribution of organizations by manufacturing segment

Industry	Number	Share (in %)
*Automotive supplier*	18	26.8
*Machinery and plant engineering*	33	49.3
*Medical technology and products*	9	13.4
*Microelectronics*	2	3.0
*Others with use of nanotechnology*	5	7.5
*Total*	**67**	**100.0**

**Fig. 4:**
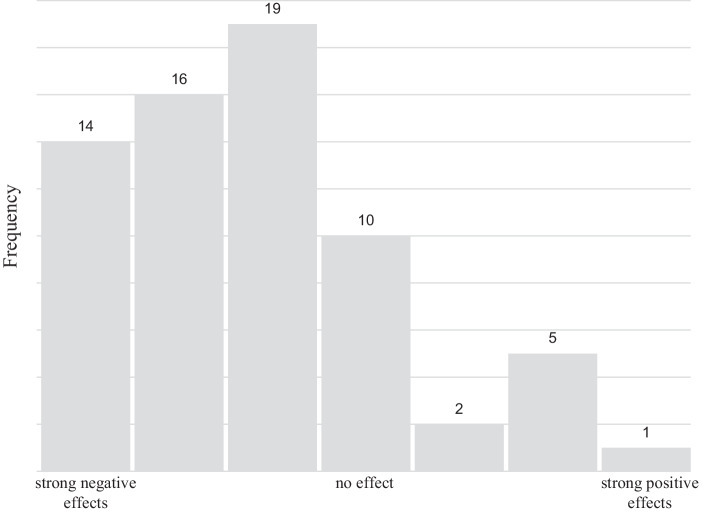
Impact of the Covid-19 pandemic in 2020

**Fig. 5:**
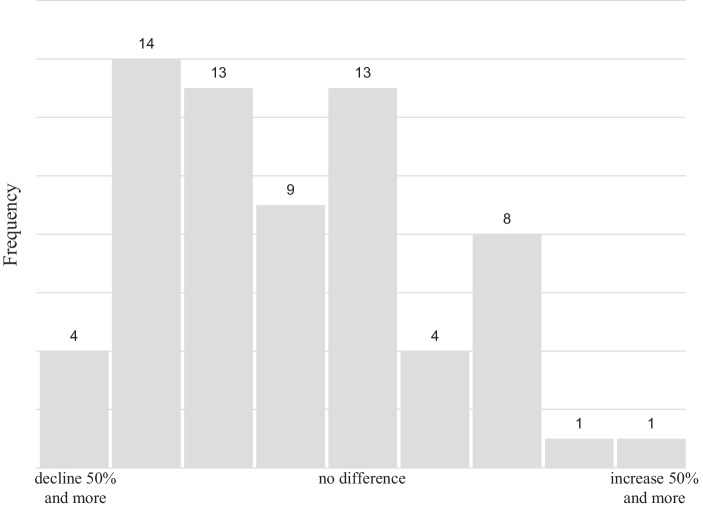
Revenue development in 2020 vs. 2019

The impact of the Covid-19 pandemic on the organization was assessed using three different dependent variables. The first and second variable are singular items answered directly by the participants. The third variable is a battery of 12 organizational items structured by the input/throughput/output logic by Slack et al. (2013). Table 5 summarizes the descriptive results while Figs. 4 and 5 show the distribution of responses on the overall impact of Covid-19 pandemic on the company in 2020 and the revenue development in 2020 compared to 2019. Most of the respondents report that their company has been severely affected by the pandemic in 2020. Looking of the overall impact of the Covid-19 pandemic on the organization in 2020 (dependent variable 1), the mean of 2.8 is significantly below the median of the scale at 4.0. Around 21% of organizations even report “strong negative impacts” (rating of 1), while only 12% of organizations report some kind of positive effect (rating between 5 and 7). Looking on the revenue development 2020 vs. 2019 (dependent variable 2), the mean of 4.0 is below the median of the scale at 5.0 as well. The majority of organizations report a revenue decline between 25% and 50% (rating of 2, 14 organizations), the second most mentioned categories are a revenue decline between 10% and 25% and no revenue change (ratings of 3 and 5, 13 organizations each). Considering the impact of the Covid-19 pandemic on different areas of the organization (dependent variable 3) it becomes evident that the area most affected by the Covid-19 pandemic is the supply chain, and the area least affected is the product range.

**Table 5:** Variables for measuring the consequences of the Covid-19 pandemic

Dependent variable	Scale	Descriptive statistics (mean)
*Overall impact of Covid-19 pandemic on organization in 2020*	7‑point Likert scale, 1: Strong negative impact 7: Strong positive impact	x = 2.8
*Revenue development 2020 vs. 2019*	9‑point Likert scale, 1: > 50% revenue decline 9: > 50% revenue growth	x = 4.0
*Impact of Covid-19 pandemic on different organizational areas:*	7‑point Likert scale, 1: Strong negative impact 7: Strong positive impact	–
Input: Suppliers	–	x = 2.2
Input: Logistics	–	x = 3.1
Input: Warehousing	–	x = 3.5
Throughput: Production	–	x = 3.1
Throughput: Human resources	–	x = 3.2
Throughput: Innovation	–	x = 4.0
Throughput: Liquidity	–	x = 3.1
Throughput: Locations/branch	–	x = 3.8
Output: Product range	–	x = 4.3
Output: Sales price	–	x = 3.6
Output: Quantity sold	–	x = 3.1
Output: Distribution	–	x = 3.1

As described above, two dimensions were assessed for the 54 items derived from the systematic literature review to measure business model resilience. The first dimension is a self-assessment of the organization. Respondents were asked on a 5-point Likert scale to what extent the item applies to their own organization. There was a tendency to judge the own organization favorably, resulting in a mean score of 3.4. In the second dimension, respondents were asked to assess whether the statement was true in terms of managing a crisis such as the Covid-19 pandemic. The overall mean of the scale of 3.5 indicates that, on average, respondents find the items well suited to assess business model resilience.

### Development of Business Model Resilience Framework

The first objective of the study was to reduce the longlist of theoretically derived factors and items to a shorter subset that is of practical relevance based on the empirical results. To achieve this, a quantitative and a qualitative approach were combined. As a first step, the items were reduced quantitatively by determining whether singular items fit into the overall scale and excluding those that did not fit. For this purpose, an item-total correlation was calculated (Churchill 1979). The item-total correlation is a common psychometric method. It is a measure of reliability. Its value indicates how good the value of a singular item fits with the value of the total scale. It is measured by the correlation of the item value with the total scale value. The higher the score, the better the agreement of the items with the scale. If the correlation drops below 0.3, the item is usually excluded from the scale (Everitt and Skrondal 2002). We applied a more conservative approach and set the cutoff value to an item-total correlation value of 0.4 (Bortz and Döring 2006). Using this threshold, we were able to exclude 29 items from the longlist of items, and 25 items remained. Restoring the mapping of the items to the theoretically derived factors, one can see that 10 of the 13 theoretically derived factors are covered by the 25 items derived from the quantitative analysis. Table 6 shows the number of items per factor included in the set of 25 items.

**Table 6:** Overview of the factors included in the resilience index based on quantitative analysis

Resilience factor	Number of items
*Corporate culture*	5
*Crisis management*	3
*Customer focus*	2
*Digitization*	4
*External collaboration*	1
*Finance*	–
*Human resources*	4
*Innovation*	1
*Leadership*	2
*Product focus*	–
*Strategy*	2
*Supply chain*	–
*Value proposition*	1

A second analysis was conducted to assess whether the quantitatively derived set of items and factors match the qualitative information provided by the respondents on the factors that were indicated as beneficial or harmful to their organizations’ performance during the Covid-19 pandemic. The respondents were asked to name up to three internal organizational aspects that have been beneficial or limiting to their management of the Covid-19 pandemic. Overall, 252 responses were given, of which 137 were beneficial and 115 were limiting aspects. Using the theoretically derived definition of business model resilience factors (see chapter 2.2.2), the free-text answers were mapped to the 13 factors. This was done independently by two researchers. Deviations from the mapping results were clarified through discussions. Tables 7 and 8 show the results for each resilience factor for both questions. A comparison of the top three most mentioned factors in the free-text responses with those factors already covered in the quantitatively derived set of items (Table 6) shows that five out of six factors are already covered. However, the Supply chain factor was mentioned 38 times as a limiting factor and is not yet included in the 25 quantitatively derived items. We therefore add further items from the longlist of the scale to cover this factor as well. In the longlist of 54 items, three items were included to cover the supply chain factor. The content of these items was compared to the free-text answers of the respondents, and two items with the greatest overlap in content were selected and added to the business model resilience scale.

**Table 7:** Beneficial factors for coping with the pandemic. Quotations have been translated analogously

Resilience factor	Number of comments	Example
*Human resources*	23	“Home office, flexible working hours”
*Strategy*	15	“New business models and markets developed”
*Crisis management*	14	“Early Covid-19 safeguards (even before government) and contingency plans”
*Customer focus*	13	“Broad customer spectrum, expansion of customer support”
*Product focus*	12	“Development of sustainable products”
*Digitization*	11	“Predominantly digitized distribution channels have proven advantageous”
*Supply chain*	11	“Diversity among suppliers”
*Value proposition*	11	“We have focused on marketing our software solutions to reduce dependencies from hardware-based solutions.”
*Culture*	9	“Regular, frequent communication with and information to employees”
*Innovation*	8	“Previous investments in R&D paid off during the pandemic”
*Finance*	6	“High equity capital”
*External collaboration*	3	“We have a strong, regional network of cooperation partners.”
*Leadership*	1	“Quick decision-making processes”

**Table 8:** Limiting factors for coping with the pandemic. Quotations have been translated analogously

Resilience factor	Number of comments	Example
*Supply chain*	38	“Bottlenecks for supplier products”
*Customer focus*	17	“Major customers with partially very restrictive measures”
*Human resources*	16	“Lack of flexibility on the part of employees”
*Finance*	12	“Weak liquidity”
*Crisis management*	11	“Regulatory requirements and many changes in quick succession”
*Value proposition*	6	“We were limited in marketing our solutions as contact with customers was hindered.”
*Product focus*	4	“We were limited by our products being focused solely at the automotive sector.”
*Strategy*	4	“Due to the crisis, planned projects were postponed or canceled.”
*Digitization*	3	“Lack of digitization”
*Leadership*	2	“Resistance of previous management to further diversification of customer structure”
*External collaboration*	1	“Our coordination efforts with customers, suppliers and authorities have increased significantly.”
*Culture*	1	“Classification of employees into vaccinated and non-vaccinated”
*Innovation*	–	–

In summary, the longlist of 54 items covering 13 factors of business model resilience was reduced to 27 items covering 11 factors by applying both a quantitative and a qualitative approach. Adding to the validity of the items derived through this approach, we assessed respondents’ answers on the second dimension of the scale (“To what extent do you consider the statement useful for dealing with crises such as the Covid-19 pandemic pandemic?”). The score for the 27 items in the final scale is 3.7, which is above the average assessment of all 54 items (x = 3.5), indicating that our methodology led to a selection of items that respondents also deemed to be particularly relevant for assessing business model resilience. Respondents’ answers on the derived set of 27 items serve as a basis for the further analysis depicted in chapter 4.3.

### Demonstration of Practical Relevance of Business Model Resilience Framework

**Fig. 6:**
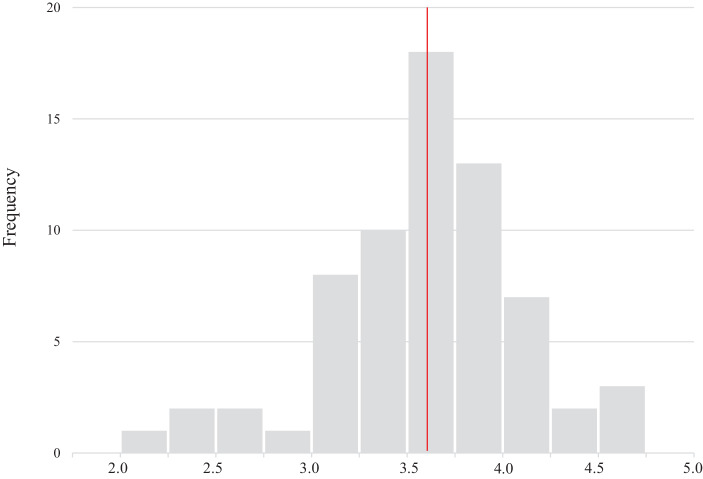
Distribution of business model resilience score (*n* = 67)

In order to determine the benefit of the business model resilience concept, we further aim to demonstrate its practical relevance in terms of organizational performance. The hypothesis in the context of this study is that organizations with high business model resilience scores have performed better during the Covid-19 pandemic than those with low scores. To test this hypothesis, we divided the sample into two groups based on their business model resilience score and compare their performance using the three different dependent variables in the study. As all items are theoretically derived to measure the concept of business model resilience, it is reasonable to aggregate all items that are part of the scale. Thus, we calculate an overall business model resilience score for each organization based on the 27 items included in the framework. Fig. 6 shows the distribution of the scores. The scores range from 2.0 to 4.7, with a mean of 3.6.

We split the total sample based on the median which is at 3.6 as well. This results in two similarly sized groups. Table 9 shows the descriptive statistics for both groups.

**Table 9:** Formation of two groups with high/low business model resilience

Business model resilience score	Descriptive statistics resilience score (mean, range, standard deviation)
*Group 1: low resilience* *(n* *=* *34)*	Mean = 3.2
	Range = 2.0 to 3.6
	Standard deviation = 0.40
*Group 2: high resilience* *(n* *=* *33)*	Mean = 4.0
	Range = 3.7 to 4.7
	Standard deviation = 0.28

To see whether these groups performed differently during the Covid-19 period, t‑tests for independent samples were conducted using the three dependent variables from the survey. First, the overall assessment of the organizations’ performance during the Covid-19 pandemic. Second, the organizations’ revenue development in 2020 compared to 2019. Third, the assessment of the impact of the Covid-19 pandemic on specific areas within the organization (see chapter 4.1 for a description of the areas assessed). For the latter, an overall score was calculated for each organization, serving as a single dependent variable. The results of the group comparisons are shown in Table 10.

**Table 10:** Comparison of the mean values of the groups in dependent variables

Dependent variable	Mean values of the groups in dependent variables	Result comparison of means (t-test for independent samples)
*Overall impact of Covid-19 pandemic on the organization in 2020*	Low resilience: × = 2.6 High resilience: x = 3.0	T = −1.2 *p* = 0.23
*Revenue development 2020 vs. 2019*	Low resilience: x = 3.6 High resilience: x = 4.4	Organizations with high resilience report less revenue decline in 2020 vs. 2019 T = 1.7 *p* = 0.08*
*Impact of Covid-19 pandemic on different organizational areas (aggregated score from 12 areas)*	Low resilience: x = 3.2 High resilience: x = 3.5	Organizations with high resilience report less negative impact on different organizational areas due to Covid-19 pandemic T = −2.8 *p* = 0.01**

Significance levels: ** p < 0.10, ** p < 0.05

In two out of three dependent variables, organizations with high scores in business model resilience report that they performed significantly better during the Covid-19 pandemic than those with low scores: They had less revenue decline in 2020 vs. 2019 and experienced a less severe impact on specific areas within the organization. Despite the difference in mean scores, the groups do not differ significantly in their assessment of the overall impact of the Covid-19 pandemic on the organization in 2020.

## Discussion

**Fig. 7:**
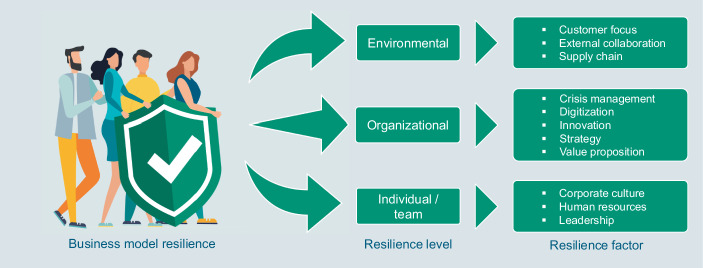
Fraunhofer IMW business model resilience framework

Although a lot of research has been conducted in the field of resilience, there is still a lack of a unified framework for business model resilience that has proven its practical relevance. This gap has become especially blatant since the Covid-19 pandemic, which triggered an intense discussion about the resilience of organizations’ business models. The objective of our paper is to close this gap. By validating findings from a systematic literature research in an empirical survey among managers and decision-makers from SMEs in Saxony, we identified 11 factors that are constitutive of business model resilience. These factors are assessed by 27 items. To provide evidence for the practical relevance of the factors, we demonstrate that organizations scoring high in business model resilience performed significantly better during the Covid-19 pandemic in 2020 than those scoring low. To provide managers as well researchers with a more tangible overview of the key components constituting business model resilience, we mapped the 11 factors derived from our research to the four levels of resilience described in chapter 2.1. The mapping was conducted following the definitions provided by the authors to describe the different levels of resilience (see chapter 2.1; Cronenberg 2020; Brink et al. 2021), as well as the definitions of the business model resilience factors that were derived from the systematic literature analysis (see chapter 2.2.2). As the factors Corporate culture, Human resources and Leadership all cover both an individual as well as a team level of resilience (Soucek et al. 2016) we did not differentiate between these two levels. As a result, the business model resilience framework shown in Fig. 7 emerges. We consider the figure a framework as it brings together insights from the literature to date to give a broader understanding of the relevant factors to consider when assessing an organization’s business model resilience, i.e. the ability of an organization to sustain its value proposition despite unexpected current and future disruptions (Imenda 2014). Having in mind the differentiation of business model resilience to other adjacent concepts (e.g. risk management, business continuity management, organizational agility) described in chapter 2 it is worthwhile reiterating the added value of the concept. Business model resilience describes the ability of an organization to sustain its value proposition despite unexpected current and future disruptions. It goes beyond other adjacent concepts in being more holistic, both vertically (by considering the individual level, the team level, the organizational level, and the environmental level; Cronenberg 2020; Brink et al. 2021) as well as horizontally (by considering all phases that an organization is going through when confronted with a disruption—from preparation to recovery; Thoma et al. 2016). Having in mind this broad scope of the concept, the framework provides significant value by providing a concise overview of the key factors that constitute business model resilience. Considering those factors both vertically as well as horizontally against the background of business model resilience is is useful for managers and decision-makers as well as for the research community. For managers and decision-makers, it is the first empirically validated framework that identifies specific organizational levers for improving business model resilience (i.e. the 11 resilience factors included in the framework). We demonstrate that from a practitioner’s perspective, it is desirable to assess and improve business model resilience. Having a high business model resilience means that it is significantly less affected by crises, both across different organizational areas and in terms of revenue development. Furthermore, by substantiating the framework with items, we provide a tool that can be used by managers and decision-makers to assess the resilience of their business models, providing insights into the current situation and allowing for derivation of improvement measures. For the research community, the main achievement of the study is that it brings together the findings from the literature by identifying the key factors for business model resilience and validating them in practice. This includes identifying the 11 factors that are now part of the business model resilience framework as well as validating them externally with organizational performance criteria during the Covid-19 pandemic. Therefore, our study is an important contribution to close the gap between theory and practice regarding the concept of business model resilience. From here, researchers can recur to the framework provided in this paper and contribute to the further investigation of a concept with high practical relevance. For example, our paper is an important contribution to the ongoing conceptual discussion of resilience in a business context (Hillmann and Guenther 2021). Identifying factors that are theoretically and empirically constitutive to the concept of resilience in a business context are an important step in developing a clear concept that can be differentiated from other adjacent managerial concepts. Furthermore, our framework provides great opportunity to link the vast body of research on resilience that has been conducted from a social-economical (Folke 2006) with a managerial perspective (i.e. through the environmental level). To support the practical relevance of the shortlisted set of items, a linear regression could have been conducted (Bortz and Döring 2006). If the derived measures for business model resilience are practically relevant, they should be able to explain a significant amount of the variance in performance indicators during the Covid-19 pandemic, such as the revenue development. Hence, further quantitative research is needed to confirm the results described in this article. Future studies should expand the scope and include organizations from other industries and regions. Last but not least, the business model resilience framework can explain the Covid-19 pandemic impacts in retrospect. Its applicability to future events that challenge the resilience of an organization’s business model remains to be demonstrated.

To increase the practical relevance of the business model resilience framework, large-scale studies should assess benchmark scores from different industries and regions. In order to increase the applicability of the results of the study, a management toolkit should be developed that is based on the framework proposed in this study and enhances it with methods to improve business model resilience. In practice, the business model resilience framework should be applied to monitor and assess the status quo of business model resilience in organizations. Based on the results, the management toolkit should provide managers and decision-makers with specific measures for the identified strengths and weaknesses and empower them to improve the resilience of their organizations.
